# *p27*-V109G Polymorphism Is Not Associated with the Risk of Prostate Cancer: A Case-Control Study of Han Chinese Men in Central China

**DOI:** 10.1155/2018/1418609

**Published:** 2018-03-20

**Authors:** Meng Zhang, Qianjun Liang, Ligang Zhang, Zongyao Hao, Jun Zhou, Li Zhang, Song Fan, Chaozhao Liang

**Affiliations:** ^1^Department of Urology, The First Affiliated Hospital of Anhui Medical University, Hefei, China; ^2^Institute of Urology, Anhui Medical University, Hefei, China; ^3^Graduate School, Anhui Medical University, Hefei, China

## Abstract

**Objective:**

We conducted an update meta-analysis aiming to verify the association between *p27*-V109G polymorphism and cancer risk, particular for prostate cancer (PCa). Then, we conducted a case-control study of Han Chinese in central China to verify the evidence-based results.

**Methods:**

Relevant studies were collected from diverse databases up to March 2017. In addition, a hospital-based (H-B) case-control study enrolling 90 PCa patients and 140 healthy controls was included to verify these evidence-based findings. Genetic risk was calculated by odds ratio (OR) with its corresponding 95% confidence interval (CI). The *p27*-V109G polymorphism was determined by MassARRAY genotyping method.

**Results:**

Finally, twenty-four published studies comprising 9627 cases and 12,102 controls were enrolled for the current meta-analysis. Overall analysis suggested that *p27*-V109G polymorphism decreased overall cancer risk in allelic contrast, heterozygote, and dominant models. When stratified analysis was conducted by ethnicity, data revealed that *p27*-V109G polymorphism was associated with a decreased cancer risk in Caucasians. Highlighted in the subgroup analysis by cancer type, we uncovered a significantly decreased risk of PCa in allelic contrast, dominant, homogeneous, and recessive models. However, in the validation case-control study, we failed to uncover a positive association between *p27*-V109G polymorphism and PCa risk. In addition, negative results were also identified when subgroup analyses were stratified by age, tumor grade, tumor stage, PSA levels, and other measurements.

**Conclusion:**

Although evidence-based results suggest that *p27*-V109G polymorphism plays a protective role in overall cancer risk, particularly for PCa, our case-control study failed to validate any association between this particular polymorphism and PCa risk.

## 1. Introduction

Prostate cancer (PCa) is the most common malignancy in the urogenital system of men and has a high lethality. In the Asian population, although PCa incidence remains at a relatively low level, it keeps increasing [1, 2]. Therefore, it is important to improve the diagnosis and treatment level of PCa. At present, like other cancers, pathogenesis of PCa is not clear [3], and lifestyle behaviors [4–6] and genetic factors [7–10] are considered as risk factors. In addition, study has shown that the incidence of PCa is about 57% gene-related [11], and of them, *p27* is one of the extensively studied genes.

Abnormal cell cycle regulation can lead to the occurrence of cancer [12]. As an important cell cycle regulatory gene, the *p27* (also known as CDKN1B) controls the cell cycle check from G1 to S transition [13]. Downregulation of the p27 expression is associated with various malignant neoplasms, including PCa [14, 15]. According to the dbSNP database, there are at least 59 mutated sites in the *p27*. Previous researches have concentrated on one of the important polymorphisms, namely *p27*-V109G (rs2066827), formed in a polymorphism at codon 109 (T>G) which caused substitution of glycine from valine and potential functional protein alteration [16]. The protein alteration, caused by *p27*-V109G polymorphism, leads to the reduction of the p27 expression, weakens negative regulation of the cell cycle, and induces tumorigenesis [15, 17].

Recently, some publications have reported the association between *p27*-V109G polymorphism and cancer risk, whereas conclusion was inconsistent, particularly for PCa. For example, some of them suggested that the *p27*-V109G polymorphism was associated with a decreased risk of cancer [18, 19], including PCa, while one research indicated that the *p27*-V109G polymorphism was not associated with PCa [20]. Therefore, in order to find out the precise relationship between this polymorphism and cancer risk (particularly for PCa), we performed an updated meta-analysis. In addition, we also conducted a hospital-based (H-B) case-control studies to further validate these evidence-based findings.

## 2. Materials and Methods

### 2.1. Updated Meta-Analysis

#### 2.1.1. Search Strategy and Study Selection

Relevant literatures were collected from PubMed, Google Scholar, Web of Science, CNKI, and Wanfang Data databases up to March 2017, using the following retrieval types: (“p27” OR “CDKN1B” OR “V109G” OR “rs2066827”) AND (“polymorphism” OR “mutation” OR “variant” OR “SNP” OR “genotype”) AND (“cancer” OR “carcinoma” OR “tumor”). Articles satisfied with the following inclusion criteria would be enrolled: (1) studies investigated the association between *p27*-V109G polymorphism and cancer risk, (2) designed as case-control, (3) genotype and allele frequencies can be extracted to calculate odds ratio (OR) and 95% confidence interval (CI), and (4) full-text studies published in English or Chinese. The major exclusion criteria were (1) case reports, case-only, or Review studies and (2) studies without raw data for *p27*-V109G polymorphism (or contacting the corresponding author still did not obtain the necessary original data). In addition, discrepancies will be resolved by consensus.

#### 2.1.2. Statistical Analysis

Statistical analyses were conducted by STATA 12.0 software (StataCorp., College Station, TX). The common measure of association across studies, ORs corresponding with 95% CIs were applied to evaluate cancer susceptibility. Pooled ORs of four genetic models were computed: allelic contrast, heterozygote, homozygote dominant, and recessive models. Chi-square test-based Q test was adopted to measure the heterogeneity across studies. In addition, the Hardy–Weinberg equilibrium (HWE) of control groups was calculated using chi-square test. The stability of results was estimated by sensibility analysis, and publication bias was estimated by Begg's funnel plot and Egger's regression test. Statistically significance was set at *P* < 0.05.

### 2.2. Case-Control Study

#### 2.2.1. Study Subjects

In this study, both patients and controls were recruited from the Department of Urology, the First Affiliated Hospital of Anhui Medical University. A total of 90 PCa patients and 140 controls were enrolled for this work between September 2014 and July 2016. Patients were newly diagnosed and pathologically confirmed as PCa, and control groups were almost age-matched, with normal prostate-specific antigen (PSA) levels (<4 ng/ml), without cancer history. Each participant signed an informed consent. We also collected relevant clinicopathologic data, as well as body mass index (BMI), systolic blood pressure (SBP), and serum PSA levels from medical record review.

#### 2.2.2. Genotyping Analysis

The genomic DNA of each participant was extracted from blood sample by using commercially DNA extraction kits (Qiagen, catalog: 51106). For each DNA sample, *p27*-V109G polymorphism was detected by MassARRAY (Sequenom, San Diego, CA). Primer was designed by Assay Design software package (Sequenom) (5′-TGCAGACCCGGAGAAAG-3′, 5′-CCGCTAACCCCGTCTGG-3′). Details of the experiment process were referring to Qin et al.'s [21] work. And results were analyzed by TYPER 4.0 software (Sequenom).

#### 2.2.3. Statistical Analyses

HWE of controls was calculated by chi-square statistics. The cancer risk, which was assessed by ORs with 95% CI, was calculated by using logistic regression models; otherwise, calculation results were adjusted for age and BMI. *P* < 0.05 was regarded as statistically significant. Differences of different clinical stages, Gleason grades, and PSA levels in PCa patients were also calculated by using the same statistic methods mentioned above. Moreover, in order to control possible confounding, logistic regression model was conducted to adjust these demographic characteristics. All statistical analyses were executed based on SPSS 17.0 version (SPSS Inc., Chicago, United States).

## 3. Results

### 3.1. Meta-Analysis

We performed an updated meta-analysis to evaluate the association between *p27*-V109G polymorphism and cancer risk. After restricting the selection process referring to the inclusion and exclusion criteria, a total of 24 articles were enrolled (Supplementary Table 1) [18, 20, 22–41]. In addition, four studies were not consistent with HWE [18, 30, 38, 40]. Publication selection process is presented in Supplementary Figure 1.

Overall, a significant decreased risk of overall cancer was identified in three genetic models (G versus T: OR = 0.859, 95% CI: 0.764–0.967, *P* = 0.012; GT versus TT: OR = 0.881, 95% CI: 0.783–0.991, *P* = 0.035; and GT/GG versus TT: OR = 0.829, 95% CI: 0.729–0.942, *P* = 0.004) (Supplementary Table 2). In the hierarchical analysis by ethnicity, data revealed that *p27*-V109G polymorphism was associated with a decreased risk of overall cancer in Caucasians instead of Asians (GT/GG versus TT: OR = 0.913, 95% CI: 0.847–0.983, *P* = 0.016). Highlighted in the subgroup analysis by cancer type, we also uncovered a significant decreased risk of PCa in four genetic models (G versus T: OR = 0.537, 95% CI: 0.365–0.789, *P* = 0.002; GT/GG versus TT: OR = 0.554, 95% CI: 0.351–0.876, *P* = 0.012; GG versus TT: OR = 0.320, 95% CI: 0.129–0.791, *P* = 0.014; and GG versus GT/TT: OR = 0.363, 95% CI: 0.149–0.886, *P* = 0.026).

In addition, sensitivity analysis suggested that overall results did not change obviously and remained robust after removing any of the individual research, which proved the stability of our results (Supplementary Table 3). Begg's funnel plot and Egger's regression test were executed to assess publication bias, and final results suggested no publication bias existed (Supplementary Figure 2).

Furthermore, in order to validate the evidence-based findings obtained above, which suggested that *p27*-V109G polymorphism was a protective factor for PCa, we further conducted a H-B case-control study of Han Chinese men in central China. Details of the case-control work were demonstrated as follows.

### 3.2. Characteristics of the Study Population in the Case-Control Study

Table 1 summarized the detailed demographic characteristics of recruited cases and controls. Mean ages of patients and controls were 73.00 ± 7.64 years (range from 48 to 87 years) and 67.66 ± 6.69 years (range from 60 to 95 years), respectively. The ages of the two groups showed a statistically significant difference (*P* < 0.001). No significant difference was uncovered in the BMI and blood pressure subgroups.

### 3.3. *p27*-V109G Polymorphism with Risk of PCa

The genotypic distributions of *p27*-V109G polymorphisms were shown in Table 2. The genotype frequency of controls was conformed to HWE (*P* = 0.565). Compared to TT genotypes, the variant TG and GG genotypes were not associated with PCa risk after adjusting for other potential covariates. The frequency of *p27*-V109G genotypes was similar between PCa cases and controls (Table 2). In addition, a negative result was also uncovered when stratified analyses were conducted by different age groups and BMI (Table 3).

For genotypic comparison, patients with different Gleason grades (based on degree of differentiation between cells, low grade < 7 and high grade > 7), clinical stages [based on international tumor-node-metastasis (TNM) system for PCa, localized and advanced], and PSA levels (PSA ≤ 20 ng/ml and PSA > 20 ng/ml) were subcategorized into 2 subgroups. However, no significant association was also observed in all these subgroups (Table 4).

## 4. Discussion

As we all know, etiology of PCa remains unclear. It has been postulated that many factors can result in malignant neoplasms. In recent decades, many researchers drew attention to the association between genetic polymorphisms and cancer susceptibility [42, 43]. Since then, SNP has been found contributed to the incidence of cancer [44], including PCa.

According to the literatures, several variants located in *p27* play an inherited role in cancer. *p27* mainly include two polymorphisms [45], called rs2066827 (109T/G) and rs34330 (−79 C/T). Some researchers have shown that *p27* polymorphism has a protective effect on cancers [18, 32, 33], while other studies proved that it did not correlate with the risk of cancer in the general population [24, 46, 47]. For this reason, Lu et al. [48] conducted a meta-analysis including 17 case-control studies enrolling 9038 cases and 11,596 controls and observed that the 109T/G TG genotype had a protective role on cancer. And Cheng et al. [49] also conducted a meta-analysis aiming to verify the association between *p27* rs34330 polymorphism and cancer risk; they suggested that rs34330 polymorphism was related to an increased risk of cancer. Here, we reconducted an updated meta-analysis containing 24 case-control studies comprising of 9627 cases and 12,102 controls and identified that *p27*-V109G polymorphism was associated with a decreased risk of cancer, which was consistent with Lu et al.'s work [48]. In addition, when stratification analysis was conducted by ethnicity, we uncovered a negative result between *p27*-V109G polymorphism and cancer risk in Asians, but it seemed to work as a protective effect in Caucasians. Moreover, in stratification analysis by cancer type, we also uncovered a significant decreased risk of PCa.

Previously, several publications have studied the association between rs2066827 polymorphism and PCa risk in different ethnicities [32, 46, 50, 51]. In 2003, Kibel et al. [19] designed a study enrolling 96 cases and 105 controls of Caucasian origin, demonstrating that this polymorphism was associated with a decreased risk of PCa. In 2015, Han et al. [18] conducted a study based on 70 cases and 70 controls in an Asian population and also found that *p27*-V109G polymorphism played a protective role in PCa risk. However, results from our case-control study were not consistent with the studies mentioned above, as well as the evidence-based results derived from meta-analysis, but consistent with Huang et al.'s [20] work, which contained 190 patients and 292 controls of Asian descent, suggesting that *p27*-V109G polymorphism was not associated with PCa. In addition, our results also suggested that no significant associations were also uncovered between this polymorphism and clinicopathologic features of PCa. Hereditary heterogeneity of subjects, different polymorphisms of *p27*, or inadequate adjustment for confounding factors can lead to the above inconsistency. Apart from these, sample size may also affect the results.

Although we have carried out a comprehensive retrieval on diverse databases, and obtained a convincible evidence-based results, which were also validated by a H-B based case-control study, there are still several limitations that should be noted in our study. First, only studies published in English or Chinese were included, which will result in potential study selection bias. Second, for PCa, they are only three case-control studies included and, of them, two generated from an Asian (Chinese) population. Although we obtained a positive association between *p27*-V109G polymorphism and PCa risk, the enrolled sample size was relatively small, which may contribute to false-positive or false-negative findings. Thirdly, we only took the *p27* genetic variant into account; other genetic factors, such as gene-gene and gene-environment interactions, can also influence cancer risk. Fourthly, for subgroup analysis, lack of adequate eligible studies may lead to insufficient statistical power, limiting the ability to identify weakly positive results. Fifthly, for the case-control study, we only made a genetic analysis of the *p27*-V109G in a Chinese population and the sample size was also limited. Moreover, the age of the cases and controls and the existing statistical significance may affect the results of the current work.

Taken together, the current work suggests that *p27*-V109G polymorphism may not be the risk factor for PCa, a result inconsistent with our meta-analysis work. Although meta-analysis is a powerful tool to cumulate and summarize the knowledge in a research field through statistical instruments, it can also be limited by the number of studies and sample sizes enrolled. Nevertheless, all these findings may require larger sample-size studies in the Chinese Han population to further validate them.

## Figures and Tables

**Table 1:** Characteristics of PCa cases and controls in a Chinese population.

Characteristic	Case	Control	*P* value^a^	OR (95% CI)
Sample size	*N* = 90	*N* = 140		
Age (years ± SD)	73.00 ± 7.64	67.66 ± 6.69	<0.001	
Age (years)			<0.001	3.715 (2.128–6.487)
≤70	34	97		
>70	56	43		
BMI			0.098	1.661 (0.910–3.030)
≤23	21	47		
>23	69	93		
Systolic blood pressure			0.787	1.076 (0.633–1.827)
<140	44	71		
≥140	46	69		

^a^Two-sided *χ* ^2^ test for the distributions between the cases and controls.

**Table 2:** Relationship between the *p27*-V109G polymorphism and PCa risk.

Genotype	Cases	Controls	*P* value^a^	OR (95% CI)	OR (95% CI)^b^
TT	77	127		1.00 (reference)	
TG	13	13	0.231	1.649 (0.727–3.742)	2.132 (0.881–5.155)
GG	0	0	—	—	—
TG/GG	13	13	0.231	1.649 (0.727–3.742)	2.132 (0.881–5.155)
*Allele*					
T	167	267	0.246	1.00 (reference)	
G	13	13		1.599 (0.724–3.532)	—

^a^Two-sided x2 test for the distributions between the cases and controls; ^b^adjusting for age and body mass index (BMI).

**Table 3:** Stratification analysis of *p27*-V109G genotypes and risk of PCa.

Group	*N* (case/control)	TT	TG/GG	*P* value^a^	OR (95% CI)
Age					
≤70	34/97	28/85	6/12	0.444	1.518 (0.521–4.421)
>70	56/43	49/42	7/1	0.100	6.000 (0.709–50.765)
BMI					
≤23	21/47	19/42	2/5	0.889	0.884 (0.157–4.973)
>23	69/93	58/85	11/8	0.157	2.015 (0.764–5.315)
Systolic blood pressure					
<140	44/71	37/62	7/9	0.627	1.303 (0.448–3.793)
≥140	46/69	40/65	6/4	0.188	2.437 (0.648–9.171)

^a^Two-sided *χ* ^2^ test for the distributions between TT and TT/GG.

**Table 4:** *p27*-V109G polymorphism and clinic pathological characteristics in Chinese patients with PCa.

Group	TT (%)	TG/GG (%)	*P* value^a^	OR (95% CI)	OR (95% CI)^b^
Clinical^c^			0.161	0.222 (0.027–1.816)	—
Localized	56	12			
Advanced	21	1			
Gleason			0440	1.600 (0.453–5.652)	1.651 (0.463–5.890)
>7	32	4			
≤7	45	9			
Total PSA (ng/ml)			0.972	0.875 (0.269–2.848)	0.978 (0.290–3.296)
>20	33	6			
≤20	44	7			

^a^Two-sided *χ* ^2^ test for the distributions between TT and TT/GG; ^b^adjusting for age and body mass index (BMI); ^c^localized, T1–2N0M0; advanced, T3–4NxMx or TxN1Mx or TxNxM1. Clinical staging according to the international TNM system for prostate cancer.

## Supplementary Materials

Supplementary Materials Supplementary Table 1: main characteristics of all studies included in the meta-analysis. Supplementary Table 2: the overall analyses of *p27-*V109G polymorphism and cancer risk. Supplementary Table 3: details of the sensitivity analyses for the *p27-V109G* polymorphism and cancer risk. Supplementary Figure 1: flow chart shows study selection process. Supplementary Figure 2: funnel plot of publication bias analysis for the associations between the *p27*-V109G polymorphism and cancer risk (GT+GG versus TT).
