# Supplementary material for: p27-V109G Polymorphism Is Not Associated with the Risk of Prostate Cancer: A Case-Control Study of Han Chinese Men in Central China
Source: Dis Markers. 2018 Mar 20;2018:1418609. doi: 10.1155/2018/1418609 (PMC5884233; doi:10.1155/2018/1418609)
Supplement: Supplementary Materials — Supplementary Table 1: main characteristics of all studies included in the meta-analysis. Supplementary Table 2: the overall analyses of p27-V109G polymorphism and cancer risk. Supplementary Table 3: details of the sensitivity analyses for the p27-V109G polymorphism and cancer risk. Supplementary Figure 1: flow chart shows study selection process. Supplementary Figure 2: funnel plot of publication bias analysis for the associations between the p27-V109G polymorphism and cancer risk (GT+GG versus TT). [file 1418609.f1.pdf]

Supplementary Information

P27-V109G Polymorphism Is Not Associated with the Risk of Prostate Cancer: A Case-Control Study of Han Chinese Men in Central China

Meng Zhang[#], Qianjun Liang[#], Ligang Zhang, Zongyao Hao, Jun Zhou, Li Zhang, Song Fan, Chaozhao Liang[*]

1. Department of Urology, The First Affiliated Hospital of Anhui Medical University, Hefei, China

2. Institute of Urology, Anhui Medical University, Hefei, China

3. Graduate School of Anhui Medical University, Hefei, China

[#] These authors contributed equally to the work.

*** Correspondence:**

Name: Chaozhao Liang;

[1] Department of Urology, the First Affiliated Hospital of Anhui Medical University, Hefei 230022, China;

[2] Institute of Urology, Anhui Medical University, Hefei 230022, China.

Tel.: +86 55162923440

Email address: liang_chaozhao@ahmu.edu.cn

Online Supplementary Material

Table S1-S3, and Figure S1-S2

Supplementary table 1. Main characteristics of all studies included in the meta-Analysis

Author	Year	Ethnicity	Cancer Type	No. of subjects cases/controls	Genotype Frequency TT/TG/GG, control-case	Genotyping method
Kibel *et al.*	2003	Caucasian	Prostate cancer	96/105	67/24/5-57/38/10	PCR
Huang *et al.*	2007	Asian	Prostate cancer	190/292	183/7/0-280/12/0	PCR-RFLP
Han *et al.*	2015	Asian	Prostate cancer	70/70	66/2/2-60/1/9	PCR-RFLP
Ma *et al.*	2006	Asian	Ovarian cancer	164/248	153/6/5-217/29/2	PCR-RFLP
Gayther *et al.*	2007	Caucasian	Ovarian cancer	1495/2584	895/524/76-1375/934/175	TaqMan
Yu *et al.*	2008	Asian	Ovarian cancer	100/95	92/5/3-95/0/0	PCR-RFLP
Jin *et al.*	2008	Asian	Ovarian cancer	234/284	219/12/3-251/31/2	PCR-RFLP
Mohamed *et al.*	2012	African	Ovarian cancer	100/100	16/33/51-25/40/35	PCR-RFLP
Li *et al.*	2004	Caucasian	Oral squamous cell cancer	713/1224	422/241/50-718/440/66	PCR-RFLP
Li *et al.*	2007	Asian	Non-small cell lung cancer	202/265	190/12/0-249/15/1	PCR-RFLP
Li *et al.*	2010	Asian	Non-small cell lung cancer	110/124	102/7/1-120/4/0	PCR-RFLP
Francisco *et al.*	2013	Mixed	Melanoma	184/173	99/75/10-66/86/21	PCR-RFLP
Daniela *et al.*	2011	Caucasian	Medullary thyroid cancer	84/90	45/38/1-56/28/6	PCR-RFLP
Liu *et al.*	2013	Asian	Hepatocellular cancer	476/547	438/38/0-490/36/21	PCR-RFLP
Guo *et al.*	2006	Asian	Esophageal cancer	299/438	281/17/1-380/53/5	PCR-RFLP
Ma *et al.*	2008	Asian	Esophageal cancer	202/265	190/12/0-249/15/1	PCR-RFLP
Sekiya *et al.*	2014	Caucasian	Endocrine tumors	517/885	223/235/59-363/406/116	Sequencing
Guo *et al.*	2006	Asian	Esophageal cancer	256/438	238/16/2-380/53/5	PCR-RFLP
Ferrand *et al.*	1996	Caucasian	Breast cancer	30/80	22/7/1-49/21/10	PCR-RFLP

MaH *et al.*	2006	Asian	Breast cancer	368/467	345/23/0-442/25/0	PCR-RFLP
Onay *et al.*	2006	Asian	Breast cancer	398/372	256/125/17-235/122/15	TaqMan
Naidu *et al.*	2007	Asian	Breast cancer	230/200	171/46/13-159/34/7	PCR-RFLP
Figueiredo *et al.*	2007	Asian	Breast cancer	1087/677	668/366/53-405/243/29	TaqMan
Driver *et al.*	2008	Caucasian	Breast cancer	2022/2179	1187/732/103-1267/796/116	TaqMan

PCR-RFLP: polymerase chain reaction–restriction fragment length polymorphism; mixed: more than two ethnicities.

Supplementary table 2. The overall analyses of *p27*-V109G polymorphism and cancer risk.

**Parameters**		**G vs. T**			**GG vs. TT**			**GT vs. TT**		
**Variables**	N	OR(95%CI)	*P*	P_h	OR(95%CI)	*P*	P_h	OR(95%CI)	*P*	P_h
All cancer	24	0.859 (0.764-0.967)	0.012	0.000	0.862 (0.659-1.128)	0.279	0.002	0.881 (0.783-0.991)	0.290	0.031
PCa	3	0.537 (0.365-0.789)	0.002	0.238	0.554 (0.351-0.876)	0.012	0.474	0.659 (0.397-1.092)	0.106	0.478
Breast cancer	6	0.985 (0.911-1.066)	0.709	0.344	0.998 (0.803-1.239)	0.984	0.468	0.973 (0.882-1.073)	0.583	0.824
Ovarian cancer	5	0.876 (0.793-0.967)	0.943	0.001	1.696 (0.678-4.243)	0.259	0.005	0.721 (0.409-1.271)	0.258	0.012
Esophageal cancer	2	0.596 (0.279-1.274)	0.182	0.094	0.309 (0.052-1.835)	0.197	0.808	0.646 (0.273-1.530)	0.321	0.073
Non-small cell lung cancer	2	1.262 (0.681-2.339)	0.459	0.149	1.242 (0.171-8.993)	0.830	0.367	1.273 (0.661-2.453)	0.470	0.371
Others	6	0.776 (0.614-0.980)	0.033	0.001	0.579 (0.303-1.106)	0.098	0.002	0.887 (0.692-1.137)	0.344	0.018
Ethnicity										
Asian	14	0.816 (0.643-1.034)	0.092	0.000	0.852 (0.493-1.473)	0.566	0.078	0.891 (0.719-1.104	0.290	0.031
Caucasian	7	0.911 (0.818-1.015)	0.090	0.043	0.824 (0.629-1.080)	0.161	0.051	0.926 (0.857-1.001)	0.053	0.199
Mixed	3	0.901 (0.443-1.833)	0.774	0.000	1.263 (0.272-5.861)	0.765	0.001	0.617 (0.306-1.244)	0.177	0.047
		**（GT+GG）vs. TT**			**GG vs.（GT+TT）**					
**Variables**	N	OR(95%CI)	*P*	P_h	OR(95%CI)	*P*	P_h			
All cancer	24	0.829 (0.729-0.942)	0.004	0.000	0.908 (0.707-1.168)	0.454	0.003			
PCa	3	0.554 (0.351-0.876)	0.012	0.474	0.363 (0.149-0.886)	0.026	0.325			
Breast cancer	6	0.919 (0.743-1.137)	0.435	0.011	1.010 (0.815-1.251	0.928	0.508			
Ovarian cancer	5	0.859 (0.523-1.412)	0.549	0.013	1.611 (0.717-3.620)	0.249	0.004			
Esophageal cancer	2	0.614 (0.268-1.409)	0.250	0.078	0.326 (0.055-1.934)	0.217	0.837			
Non-small cell lung cancer	2	1.272 (0.671-2.414)	0.461	0.238	1.220 (0.168-8.868)	0.844	0.374			

Others	6	0.810 (0.638-1.029)	0.084	0.015	0.637 (0.347-1.166)	0.144	0.004
Ethnicity							
Asian	14	0.803 (0.640-1.007)	0.057	0.005	0.867 (0.504-1.491)	0.606	0.081
Caucasian	7	0.913 (0.847-0.983)	0.016	0.104	0.859 (0.667-1.107)	0.240	0.066
Mixed	3	0.757 (0.360-1.594)	0.464	0.011	1.307 (0.377-4.529)	0.673	0.003

N= number of studies involved; OR= Odd ratio, 95%CI= 95%confidence interval; *P* =*P*-Value; P_h = *P*-value of Q-test for heterogeneity test.

Supplementary table 3. Details of the sensitivity analyses for the *p27-V109G* polymorphism and cancer risk

**Study Omitted**	**G vs. T**		**GG vs. TT**		**GT vs. TT**		**（GT+GG）vs. TT**		**GG vs. (GT+TT）**	
	**Estimate**	**95%CI**	**Estimate**	**95%CI**	**Estimate**	**95%CI**	**Estimate**	**95%CI**	**Estimate**	**95%CI**
Kibel *et al.*	0.874	0.776-0.984	0.888	0.676-1.167	0.894	0.795-1.001	0.843	0.741-0.958	0.928	0.717-1.200
Huang *et al.*	0.858	0.761-0.967	0.862	0.659-1.128	0.880	0.780-0.993	0.827	0.726-0.943	0.908	0.707-1.168
Han *et al.*	0.873	0.778-0.980	0.896	0.688-1.166	0.879	0.780-0.990	0.836	0.735-0.950	0.942	0.737-1.204
Ma *et al.*	0.863	0.765-0.973	0.838	0.642-1.094	0.898	0.803-1.003	0.839	0.738-0.955	0.886	0.691-1.345
Gayther *et al.*	0.856	0.750-0.978	0.887	0.660-1.191	0.879	0.768-1.007	0.826	0.714-0.956	0.935	0.710-1.232
Yu *et al.*	0.856	0.763-0.961	0.850	0.651-1.109	0.878	0.783-0.986	0.825	0.728-0.935	0.897	0.699-1.152
Jin *et al.*	0.868	0.770-0.978	0.850	0.647-1.117	0.897	0.800-1.006	0.841	0.739-0.956	0.896	0.694-1.157
Mohamed *et al.*	0.836	0.746-0.938	0.813	0.626-1.057	0.874	0.775-0.985	0.814	0.717-0.924	0.857	0.671-1.095
Li *et al.*	0.843	0.743-0.958	0.822	0.618-1.093	0.873	0.766-0.995	0.815	0.710-0.936	0.868	0.665-1.133
Li *et al.*	0.857	0.760-0.967	0.865	0.659-1.136	0.877	0.777-0.990	0.825	0.724-0.940	0.911	0.706-1.176
Li *et al.*	0.852	0.758-0.957	0.854	0.652-1.119	0.875	0.778-0.984	0.821	0.723-0.932	0.901	0.699-1.160
Francisco *et al.*	0.880	0.783-0.990	0.924	0.712-1.200	0.900	0.801-1.012	0.849	0.747-0.964	0.958	0.745-1.230
Daniela *et al.*	0.851	0.754-0.961	0.881	0.674-1.151	0.867	0.772-0.973	0.815	0.716-0.927	0.930	0.726-1.192
Liu *et al.*	0.880	0.783-0.989	0.893	0.695-1.147	0.869	0.770-0.981	0.832	0.729-0.951	0.939	0.745-1.184
Guo *et al.*	0.882	0.787-0.989	0.877	0.669-1.148	0.904	0.809-1.010	0.851	0.751-0.963	0.922	0.716-1.187
Ma *et al.*	0.857	0.760-0.967	0.865	0.659-1.136	0.877	0.777-0.990	0.825	0.724-0.941	0.911	0.706-1.176
Sekiya *et al.*	0.851	0.748-0.968	0.857	0.633-1.162	0.873	0.768-0.993	0.820	0.714-0.942	0.905	0.680-1.204
Guo *et al.*	0.875	0.778-0.984	0.867	0.659-1.141	0.900	0.802-1.009	0.845	0.743-0.960	0.912	0.705-1.180
Ferrand *et al.*	0.868	0.771-0.977	0.880	0.673-1.151	0.882	0.782-0.995	0.834	0.732-0.949	0.925	0.720-1.189

Ma H *et al.*	0.851	0.754-0.960	0.862	0.659-1.128	0.872	0.773-0.984	0.819	0.718-0.933	0.908	0.707-1.168
Onay *et al.*	0.850	0.750-0.963	0.847	0.637-1.127	0.875	0.771-0.993	0.820	0.716-0.939	0.895	0.685-1.169
Naidu *et al.*	0.841	0.747-0.948	0.831	0.632-1.094	0.868	0.770-0.979	0.811	0.712-0.922	0.882	0.681-1.141
Figueiredo *et al.*	0.848	0.746-0.964	0.836	0.624-1.119	0.875	0.768-0.997	0.844	0.739-0.964	0.882	0.671-1.159
Driver *et al.*	0.844	0.738-0.965	0.840	0.614-1.148	0.866	0.758-0.991	0.814	0.707-0.937	0.889	0.662-1.192

95%CI= 95%confidence interval

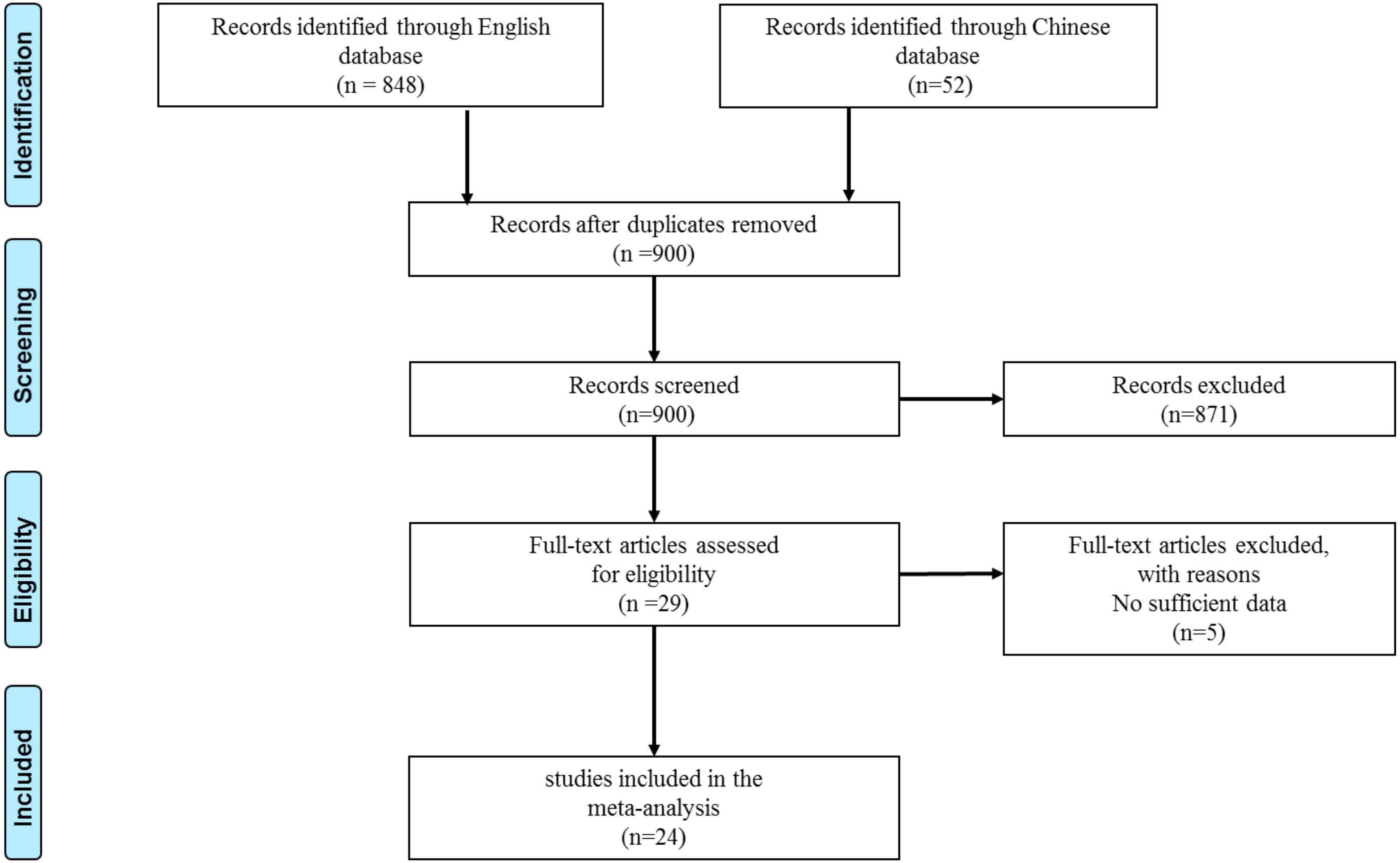

Supplementary figure 1. Flow chart shows study selection process.

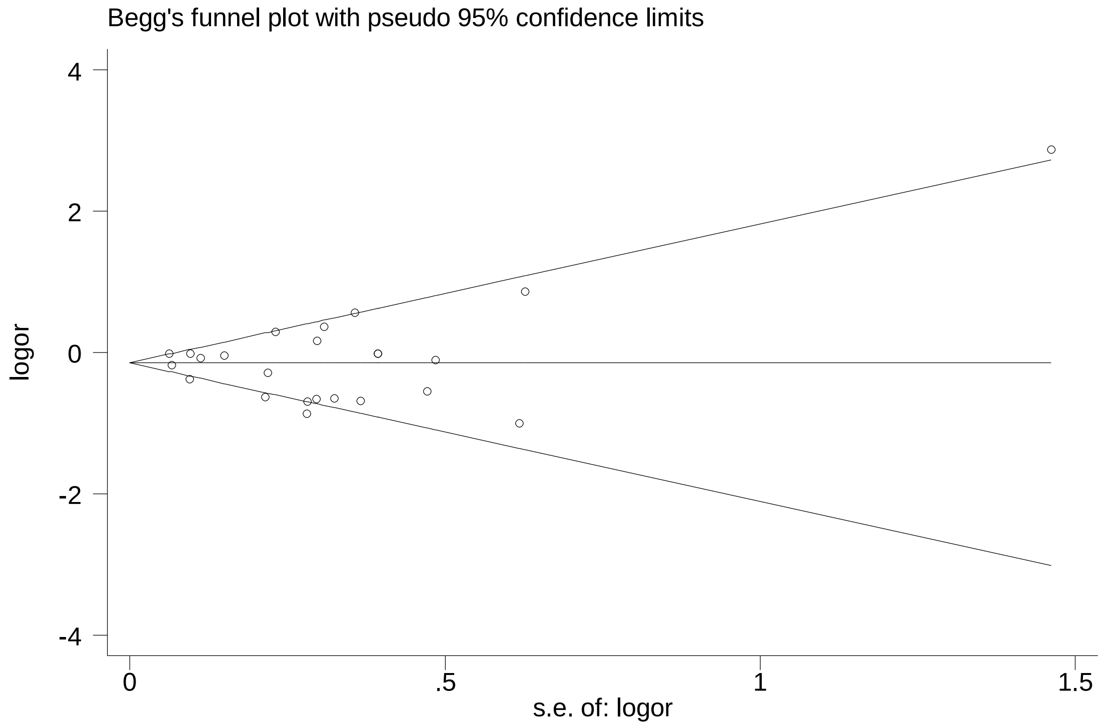

Supplementary figure 2. Funnel plot of publication bias analysis for the associations between the *p27*-V109G polymorphism and cancer risk (GT+GG vs. TT).
